# Microtubular and Nuclear Functions of γ-Tubulin: Are They LINCed?

**DOI:** 10.3390/cells8030259

**Published:** 2019-03-19

**Authors:** Jana Chumová, Hana Kourová, Lucie Trögelová, Petr Halada, Pavla Binarová

**Affiliations:** Institute of Microbiology of the Czech Academy of Sciences, Vídeňská 1083, 142 20 Prague, Czech Republic; jana.chumova@biomed.cas.cz (J.C.); kourova@biomed.cas.cz (H.K.); lucie.trogelova@biomed.cas.cz (L.T.); halada@biomed.cas.cz (P.H.)

**Keywords:** gamma-tubulin, nucleation, nuclear functions, filaments, lamins, SUN proteins, mechanosensing

## Abstract

γ-Tubulin is a conserved member of the tubulin superfamily with a function in microtubule nucleation. Proteins of γ-tubulin complexes serve as nucleation templates as well as a majority of other proteins contributing to centrosomal and non-centrosomal nucleation, conserved across eukaryotes. There is a growing amount of evidence of γ-tubulin functions besides microtubule nucleation in transcription, DNA damage response, chromatin remodeling, and on its interactions with tumor suppressors. However, the molecular mechanisms are not well understood. Furthermore, interactions with lamin and SUN proteins of the LINC complex suggest the role of γ-tubulin in the coupling of nuclear organization with cytoskeletons. γ-Tubulin that belongs to the clade of eukaryotic tubulins shows characteristics of both prokaryotic and eukaryotic tubulins. Both human and plant γ-tubulins preserve the ability of prokaryotic tubulins to assemble filaments and higher-order fibrillar networks. γ-Tubulin filaments, with bundling and aggregating capacity, are suggested to perform complex scaffolding and sequestration functions. In this review, we discuss a plethora of γ-tubulin molecular interactions and cellular functions, as well as recent advances in understanding the molecular mechanisms behind them.

## 1. Introduction

γ-Tubulin belongs to the eukaryotic clade of tubulins [1] and both human and plant Arabidopsis γ-tubulins show the highest similarity—on the sequence and structural levels—to β-tubulins and bacterial BTubA and BTubB of *Prosthecobacter dejongeii* [2]. Archaeal tubulins (called artubulins), encoded in the genomes of Thaumarchaeota, showed the highest similarity to eukaryotic tubulin sequences, in particular, to γ-tubulins [3]. It is known that α/β-tubulins exist in different isotypes with spatially and temporally regulated expressions and are altered by various posttranslational modifications [4]. γ-Tubulin is expressed from two gene duplicates in the majority of eukaryotes and their functional redundancy in microtubule nucleation was suggested [5,6]. However, mouse γ-tubulin-2 is primarily expressed in the brain and knockout analysis showed that its impaired function affects the circadian rhythm and behavior [7]. The accumulation of γ-tubulin-2 in neurons under oxidative stress points to its role in stress response [8]. Multiple charge variants of γ-tubulin detected by 2D-electrophoresis suggest the posttranslational modifications of the molecule [9,10,11]. γ-Tubulin is phosphorylated in a cell-cycle-dependent manner [12,13] and monoubiquitinated by a ubiquitin-ligase complex BRCA1/BARD1 [14]. Understanding the usage of isotypes and the posttranslational modifications of γ-tubulin in the differentiation or under stress will help us get a better insight into the cellular functions of the protein in the health and disease spheres. γ-Tubulin is known to exist in a variety of molecular forms, ranging from low-molecular-mass complexes up to several MDa-assemblies. In this review, we discuss the recent advances that have contributed to our understanding of a high heterogeneity of the molecular forms of γ-tubulin and its numerous protein interactions and functions reported across eukaryotes. 

## 2. Functions of γ-Tubulin with Microtubules and Its Interaction with Other Cytoskeletal Systems

As microtubule nucleation has been widely discussed in comprehensive review articles over the last few years [15,16], we provide a short overview concerning γ-tubulin function in centrosomal and non-centrosomal nucleation pathways and other γ-tubulin roles with cytoskeletons. γ-Tubulin ring complexes (γTuRC) built of tetrameric γ-tubulin small complexes (γTuSCs) and of other specific components (such as GCP4-6 proteins) serve as templates for microtubule nucleation [17]. The role of γ-tubulin in the nucleation of microtubule was also demonstrated in plants where centrosomes are absent in all somatic and gametic cells [18,19]. γ-Tubulin complexes with GCPs are suggested to nucleate plant microtubules from acentrosomal dispersed sites localized with membranes, especially with the nuclear envelope and with preexisting microtubules [20,21]. Plant homologs of all GCPs components of γTuRC were copurified with γ-tubulin from Arabidopsis [2]. A higher abundance of γ-tubulin in acentrosomal plant cells, compared to centrosome equipped animal cells, enables us to characterize the nuclear γ-tubulin pool and to demonstrate γ-tubulin with the centromeric/kinetochore region of chromosomes. γ-Tubulin is present in nuclei at the G1/S and G2/M phase of the cell cycle and accumulates in the centromeric/kinetochore region before the nuclear envelope breakdown and its role in nucleation and/or in the capturing of the kinetochore microtubules of the acentrosomal spindle was suggested [21,22]. γ-Tubulin complexes promote together with the nuclear pore complex NUP107-160 the kinetochore-driven microtubule nucleation in animal cells in a Ran GTPase dependent manner [23].

Non-centrosomal nucleation of microtubules from dispersed sites is not only found in higher plants but also in large animal cells and, often, centrosomal and acentrosomal nucleation is performed in the same cell [24]. In additions to GCPs, other proteins such as augmins [25,26], centrosomin-domain containing proteins [27] and membrane-linked proteins are also required for the assembly of microtubule nucleation capable units. Augmin-dependent and Ran GTPase-dependent microtubule nucleation on preexisting microtubules or on chromatin contribute to the mitotic spindle assembly in both centrosome-equipped and acentrosomal cells [24,28]. In contrast to animal cells, in plants, the function of augmins is not restricted to mitosis [29]. There are also differences in the spectra of γ-tubulin interactors depending on the presence or absence of centrosomes. For example, the centrosomin domain-containing proteins (CDK5RAP and MTO1/2) play a role with γ-tubulin in centrosome-equipped metazoa and fungi [27,30] but they are absent in acentrosomal plant cells. Membrane-driven microtubule nucleation is conserved in eukaryotes. The Golgi apparatus is a well-established microtubule nucleation site in animal cells [31] and large heterogeneous complexes of γ-tubulin-associated with membranes promote microtubule nucleation in plants [32].

Nuclear export machinery is involved in microtubule nucleation from non-centrosomal sites in *S. pombe.* Exportin CRM1 mediates in the Ran GTPase dependent manner, docking the MTO1/2 complex and γ-tubulin complexes to the nuclear pore [33]. The catalytic subunit of DNA polymerase is transported from the nuclei by CRM1 and it interacts with γTuRCs and inhibits acentrosomal microtubule nucleation at the Golgi membranes in mammalian cells [34,35]. On the other hand, microtubule-associated TPX2 protein is transported in Arabidopsis cells via the importin/RanGTP pathway to nuclei, where it promotes the formation of intranuclear microtubules together with γ-tubulin [36]. Ran GTPase and exportins were identified by LC MALDI-TOF MS/MS to be among the proteins copurified with γ-tubulin from Arabidopsis extracts in our experiments (Appendix A). All these data suggest that the nucleocytoplasmic transport of microtubule-associated proteins and chromatin-associated factors is important in the coordination of microtubule dynamics and chromatin organization. 

In addition to its function in microtubule nucleation at the minus end of microtubules, γ-tubulin also assists in stabilizing the growing ends of microtubules by loading the microtubule plus end proteins [37]. γ-Tubulin may stabilize the growing ends of microtubules by closing the seam of the nascent microtubules [38]. Stabilization of the nascent ‘nuclei’ at the growing ends of microtubules through interaction with TPX2 or with other MAPs has been recently suggested to promote nucleation efficiency [16]. Whether γ-tubulin may contribute to the microtubule nucleation in a similar way through the stabilization of the plus ends of microtubules is not clear. 

Apart from its well-established functions with microtubules, γ-tubulin interacts with actin and the Arp2/3 complex and provides a potential link between the microtubular and actin cytoskeleton [39]. The interaction of γ-tubulin with intermediate filaments was also reported [40,41]. γ-Tubulin contributes to the organization of the Golgi apparatus [42] and mitochondria [43], and participates in an inter-talk between endosomes and spindle organizations [44].

## 3. γ-Tubulin Has an Intrinsic Ability to Assemble Filaments with a Bundling and Aggregating Capacity

The interactions of γ-tubulin with GCPs and proteins with regulatory, targeting, or other functions in microtubule nucleation presumably present a source of size variability of γ-tubulin complexes. Large molecular assemblies of γ-tubulin apparently not involved in microtubule nucleation were reported from different eukaryotes and experimental systems [10,11,32]. One of the most important tasks for understanding multiple γ-tubulin cellular functions has been the characterization of large protein molecular assemblies. Several studies demonstrating γ-tubulin oligomerization and polymerization have been published over the last few years [2,41,45,46]. We found that plant and human γ-tubulins form protofilaments and higher order fibrillar assemblies in vitro [2]. Proteins of γ-tubulin complex GCPs are not essential for the assembly of either plant or human γ-tubulin filaments in vitro and the ability to assemble oligo/polymers was suggested to be an intrinsic property of γ-tubulin molecule. γ-Tubulin filaments align by lateral contacts to form double protofilaments, longer filament bundles and clusters/aggregates.

The preservation of the surfaces of αβ-tubulin for longitudinal and lateral interactions in human γ-tubulin was shown previously by the homology modeling and the ability of γ-tubulin to form dimers, oligomers and polymers was predicted [47]. Our structural studies showed that human and plant γ-tubulin molecules are conserved on the sequence and structural level, including the longitudinal and the lateral interaction surfaces of the molecule. Additionally, residues involved in GTP binding are conserved in human and plant γ-tubulin and differ from those found in GTP hydrolyzing α- and β-tubulins and prokaryotic tubulins [2]. Our data suggest that γ-tubulin preserves the characteristics of prokaryotic and eukaryotic tubulins. Dimers are formed only from properly folded endogenous γ-tubulin [2,9,48], while they are not observed for γ-tubulin produced in the baculovirus expression system or those translated in vitro [48,49,50]. The co-purification of CCT chaperones and the presence of the CCT binding site suggest the chaperone-assisted folding for γ-tubulin [2,46]. Depending on the functionality of the CCT chaperonin, either γ-tubulin aggregates or filaments are formed from human γ-tubulin [51]. In contrast to the requirements of γ-tubulin for the chaperone-dependent folding, which is characteristic of eukaryotic tubulins [46], we found that similar to prokaryotic tubulins, γ-tubulin tolerates a broader range of conditions to assemble into polymers in vitro [52]. The assembly of the double-stranded filaments we observed for plant and human γ-tubulins [2] offer higher stability compared to the single filaments. Moreover, the assembly of tubulin filaments with a bundling capacity typically found in prokaryotes minimizes the requirements for the subunit and energy supply in comparison with the formation of microtubules [53]. 

Fibrillar forms of γ-tubulin were also demonstrated in animal cells. Fine γ-tubulin filaments (called strings) with a diameter of 4–6 nm [41] correspond to the diameter of γ-tubulin filaments observed in our experiments [2]. γ-Tubulin strings localized with the nuclei span from the cytoplasm into the nuclei and show a broad distribution with membranes, mitochondria and the pericentriolar matrix. Additionally, their role in cellular processes is discussed [41]. Similarly, in acentrosomal Arabidopsis cells, fibrillar γ-tubulin was localized at all sites of the dispersed γ-tubulin distribution in the cytoplasm, with the mitotic microtubular arrays with membranes, was enriched with the nuclear envelope before mitosis and was present in the nuclei. Our data indicated that fibrillar structures with fluorescent maxima observed in cells are formed from short fine filaments at sites of local γ-tubulin enrichment [2] as observed for the filament-forming prokaryotic tubulin FtsZ [54]. 

Plant and human γ-tubulin filaments or strings [2,41,45,46] differ from filamentous structures assembled in vitro from small complexes of γ-tubulin with GCPs of *Saccharomyces cerevisiae* with the assistance of centrosomin domain-containing protein Spc110 [55]. Higher order fibrillar assemblies of γ-tubulin (called γ-tubules)—composed of γ-tubulin, GCPs and pericentrin—were detected in human cells [45]. γ-Tubules are more robust compared to γ-tubulin filaments—with a diameter of 22 nm—and are observed more frequently in non-dividing cells. γ-Tubules inter-win often with microtubules and also interact with vimentin and actin [45]. Rod-like structures positive for γ-tubulin were present mostly in the non-dividing cells in nuclei and the perinuclear area in Arabidopsis [2]. Compared to the fine fibrillar structures of γ-tubulin, robust γ-tubulin rods were also positively labeled for α-tubulin and actin. Our STED analyses suggested that the ability of γ-tubulin to oligomerize and aggregate might be behind the rod formation. Linear inclusions positive for γ-tubulin and actin observed in nuclei and the perinuclear area in mature neurons were suggested to serve as a supply of the centrosomal material in non-dividing cells [56]. 

## 4. Functions of γ-Tubulin in the Cell Cycle Regulation and in Nuclear Processes

The microtubule-independent role of γ-tubulin in the cell cycle was reported from different eukaryotes. Coordination between mitosis and cytokinesis is lost in γ-tubulin conditional mutants of *S. pombe* and in *Aspergillus* [57,58]. The microtubule-independent function of γ-tubulin in the regulation of the cell division in *Aspergillus* is performed through the APC complex [59]. Defects of the anaphase/telophase transition were also observed in Arabidopsis cells with reduced γ-tubulin levels [20]. The presence of γ-tubulin in the nuclei of plant and animal cells [60,61] suggests its function in nuclear processes. The proteins of DNA repair and DNA damage checkpoints are present in centrosomes [62,63]. Well-defined complexes of γ-tubulin with DNA repair protein BRCA1/BARD and with the Ola1 protein are localized with centrosomes where they most likely suppress centrosome amplification [64]. The activation by CDK1 and the association with BRCA1 are required for the loading of γ-tubulin to microtubules and the transportation of the BRCA1/γ-tubulin complex to nuclei [65]. Formation of a complex of γ-tubulin with Rad51 is observed under the DNA stress and suggests nuclear function of γ-tubulin in a DNA damage response [61]. The interaction of γ-tubulin and the GCP2 and GCP3 proteins with tumor suppressor C53 in nucleoli is proposed to modulate the C53 tumor suppressor activity in DNA damage checkpoints [66]. Multiple components of the DNA damage response pathway are linked to centrosomes and the genotoxic stress strongly affects the centrosome organization [67]. However, whether γ-tubulin interlinks the nuclear and centrosomal DNA response pathways is not known. In addition to its functions in response to DNA damage, genetic evidence was provided for the function of γ-tubulin in chromatin remodeling [68]. Furthermore, nuclear γ-tubulin forms a complex with the E2F1 transcription factor and functions at the G1/S checkpoint by controlling the E2F transcriptional activity [69]. 

γ-Tubulin is a platform for a number of signaling molecules including the cell cycle and DNA damage signaling [12,13,14] and stress response MAP kinases [70]. γ-Tubulin is overexpressed in many types of tumors [71] and overexpressed γ-tubulin interacts with the class III β-tubulin isotype that is specifically overproduced in glioma tumor cells [72]. γ-Tubulin and the tumor suppressor retinoblastoma RB1 protein negatively regulate each other’s expression as a high level of γ-tubulin was observed in cells with an impaired function of RB1 while RB1 was overexpressed in γ-tubulin mutants. The lethal effect of γ-tubulin depletion in tumors with non-functional RB1 proposes γ-tubulin as a target of cancer therapy [73]. Nuclear and cell cycle related functions of γ-tubulin and its overexpression and redistribution in cancer cells suggest this protein to be an important factor in tumorigenesis [71]. γ-Tubulin functions in cellular homeostasis and its impact on disease development are reviewed in Reference [74]. The regulated polymerization of coiled-coil protein SPD-5 is essential for the scaffold formation in the pericentriolar matrix [75]. γ-Tubulin filaments were also observed in the pericentriolar matrix [41] as well as in the nuclei and in other cellular compartments [2,74]. Whether the ability of γ-tubulin to assemble filaments provide a platform for the scaffolding/sequestration functions of γ-tubulin in the centrosome-equipped and acentrosomal cells is an interesting question to be addressed. 

## 5. γ-Tubulin Interacts with Lamin and the Interaction Is Required for Regular Nuclei Organization

It is most likely the fibrillar nature of γ-tubulin that is behind its interaction with other cytoskeletal systems: with microtubules [37], actin cytoskeleton [39] and with intermediate filaments [40,41]. Rod-like intranuclear inclusions containing γ-tubulin are present in neuronal and retinoblastoma cells [56,76], as well as in plant cells [2]. γ-Tubulin positive nuclear inclusions are closely associated with lamins in cancer cells [56]. 

γ-Tubulin recruits lamin B to the nuclear envelope and the interaction of γ-tubulin with lamin was required for proper nuclear organization. The meshwork of fibrillar γ-tubulin in the vicinity of nuclei was suggested to support the lamina and the organization of the nuclear envelope [41]. Lamins and associated proteins localized at the inner surface of the nuclear envelope interact in a dynamic manner with chromatin. Their role in the organization of chromatin domains, in transcription, replication, and DNA repair is known (reviewed in Reference [77]). We can presume that its interaction with lamins might be behind some of γ-tubulin’s nuclear functions. 

## 6. γ-Tubulin, Proteins of the Nuclear Envelope and LINC Complexes

Genes for lamins are not present in plants and plant lamina is not well characterized. The Nuclear Matrix Constituent Proteins (NMCPs) of higher plants contain long coiled-coil domains and are functional analogs to lamins, although they show no sequence similarity [78]. Scanning electron microscopy SEM analyses showed the fibrillar network underlying the inner nuclear envelope in plant cells and the term plamina/plant lamina was introduced [79]. With the current knowledge available, it is only a matter of speculation whether fibrillar γ-tubulin found in the plant nuclei [2] interacts with plant lamina. 

The LINC complex (LInker of Nucleoskeleton and Cytoskeleton) connects the nuclear lamina with the cytoskeleton and contributes to cellular rigidity, nuclear positioning, chromosome organization, mechanotransduction, and cell migration [80,81]. In contrast to lamins, the SUN (Sad1/UNC84) proteins are evolutionary conserved. In animals, SUN proteins localize to the inner nuclear membrane and their C-terminal domain interacts with KASH proteins at the perinuclear space while the N-terminal nucleoplasmic domain binds to the nuclear lamina [82]. The interaction of SUN proteins with lamins is regulated by the cell cycle regulatory phosphorylation [83]. SUN1,2 couple with the nuclear envelope and centrosome during nuclear migration [84] and have a role in the nuclei positioning mediated through the microtubular or actin cytoskeleton [85]. The SUN protein is also involved in chromatin remodeling during sperm head maturation [86] and in DNA repair [87]. The LINC-dependent chromosome positioning is conserved among eukaryotes. The SUN domain-containing protein Sad1 of *S. pombe* is tethered to telomeres via meiosis-specific proteins and recruits γ-tubulin complexes to telomeres, forming the microtubule-organizing center telocentrosome. Microtubules nucleated from the telocentrosomes and the molecular motors gather meiotic telomeres together and ensure bouquet-dependent homologous chromosome pairing [88]. The association of SUN1 with the telomeres in meiosis is required for homologous chromosome pairing during mice gametogenesis [89]. Plant SUN proteins were localized to specific regions of the nuclear envelope in plant meiosis and their function in organizing telomeres to ensure homologous chromatid recombination and proper progression through meiosis was suggested [90,91]. 

Plant homologs of the SUN1,2 proteins, similarly to their animal counterparts, localize with the inner nuclear envelope and have a role as nucleoskeletal anchors in maintaining nuclear organization [92,93]. In SUN mutants of Arabidopsis, the nuclei of differentiated cells lost developmentally specific morphology and became round shaped with a morphology typical for meristematic cells [94]. Plant KASH proteins, structurally different from the KASH proteins of animals, were shown to be involved in anchoring the plant RanGAP and/or myosin and actin to the nuclear envelope and to be involved in the control of nuclear positioning and movement [95,96]. Considering the connection of SUN to nuclear lamina, the interaction partners of plant SUNs on the nuclear side of the nuclear envelope are less characterized compared to KASH proteins. Arabidopsis homologs of Nuclear Matrix Constituent Proteins CRWN (Crowded Nuclei) and NEAP (Nuclear Envelope-Associated Protein) have been shown to bind SUN in Arabidopsis [97,98]. 

γ-Tubulin shows a dispersed distribution in several cellular compartments regulated in a cell-cycle-dependent manner in acentrosomal plant cells [18,19]. As we demonstrated previously by superresolution microscopy STED, fibrillar γ-tubulin is localized at the cytoplasmic side of the nuclear envelope partially with perinuclear microtubules and also in the nuclei of interphase cells [2]. Similarly, fibrillar γ-tubulin was demonstrated with nuclei in animal cells [41]. The nuclear envelope presents the most important microtubule nucleation site in the acentrosomal plant cells where perinuclear microtubules contribute substantially to the microtubular cytoskeleton organization, but it is also the main microtubule organizing center in muscle cells. LINC protein nesprins were shown to recruit centrosomal proteins and to regulate microtubule nucleation from the nuclear envelope and the nuclei positioning in the myotubes [99]. The inner nuclear envelope protein (Samp1) interacts with γ-tubulin and with SUN1 [100,101]. It was suggested that Samp1 recruits γ-tubulin from the fenestrated nuclear envelope during nuclear envelope breakdown to the spindle microtubules. In the spindle, the complex of γ-tubulin and Samp1 together with augmins may contribute to the microtubule nucleation. 

**Figure 1:**
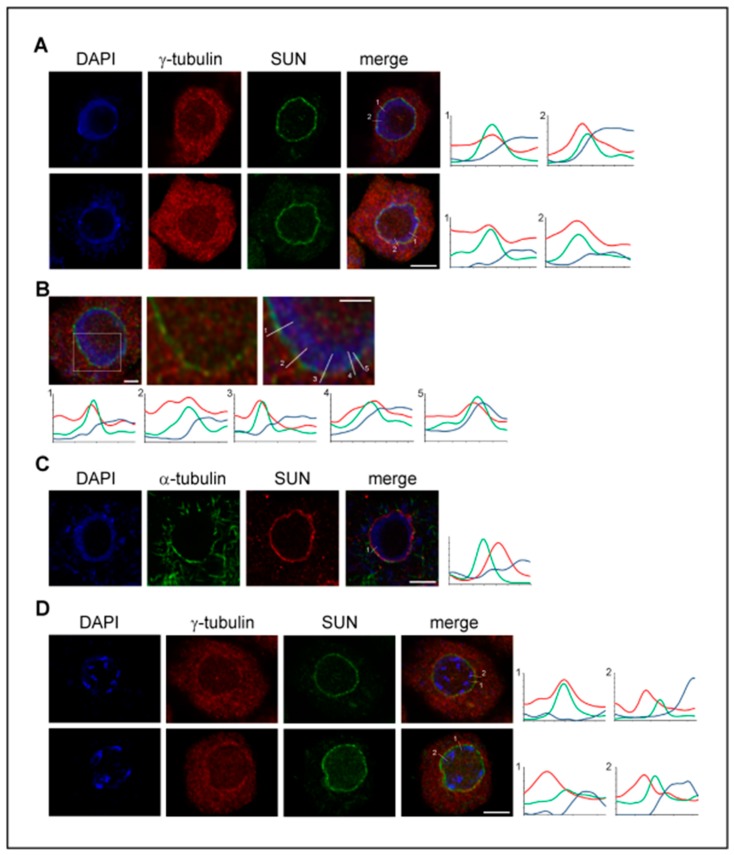
γ-Tubulin partially colocalizes with SUN1,2 proteins and is present at both the nuclear and cytoplasmic sides of the nuclear envelope. (**A**) Representative images of γ-tubulin (red) and SUN (green) localization in the interphase meristematic cells of Arabidopsis root tips obtained by confocal laser scanning microscopy equipped with the super-resolution module (CM-SR, see Appendix B). Intensity profiles show the signal of γ-tubulin and SUN measured across the nuclear envelope. (**B**) Representative CM-SR images of the nucleus immunolabeled for γ-tubulin (red) and SUN (green). Close-up view of the nucleus with a representative measurement of the intensity profiles shows the partial colocalization of γ-tubulin with the inner nuclear envelope decorated with SUN. (**C**) Representative CM-SR images with the immunolocalization of α-tubulin (green) and SUN (red) in the interphase meristematic cell. The intensity profile shows distinct maxima of α-tubulin and SUN along the measured line. (**D**) Representative CM-SR images of γ-tubulin and SUN localization during late G2 in cells with pre-mitotically condensed chromatin. The intensity profiles show γ-tubulin accumulation on the cytoplasmic side of the nuclear envelope and also the partial colocalization of γ-tubulin with SUN, similar to interphase nuclei. The intensity profile along the line (**A**–**D**): x-axis shows the length in µm measured from the cytoplasmic side (marked by number); y-axis shows the relative intensity. Scale bars: 5 µm (**A**,**C**,**D**), 2 µm (**B**).

In spite of the fact that microtubule nucleation from the nuclear envelope and from pre-existing spindle microtubules is of high importance in the plant cells where centrosomes are absent, the data on the possible interaction and function of γ-tubulin with LINC/SUN/KASH proteins are not yet available. Therefore, we analyzed the localization of γ-tubulin with SUN proteins. A γ-tubulin signal was found with the inner nuclear envelope decorated by the anti-SUN1,2 antibody and the partial colocalization of both proteins was supported by the measurement of the maximum intensity profiles (Figure 1A,B). Compared to γ-tubulin, the analyses of the α-tubulin immunolabeling of microtubules on the cytoplasmic side of the nuclear envelope and the SUN signal with the inner nuclear envelope showed, as expected, distinct maxima of intensity (Figure 1C). γ-Tubulin became enriched with the outer nuclear envelope at the late G2 phase when the intense nucleation of perinuclear microtubules occurred. However, the partial colocalization of γ-tubulin with SUN proteins at the nuclear side of the nuclear envelope was still observed (Figure 1D). 

The interaction of γ-tubulin with the SUN proteins indicated in our experiments is not completely unexpected as the functions of SUN proteins and γ-tubulin partially overlap. SUN [82] and γ-tubulin [41] interact with lamins and the interaction is required for proper nuclear organization. γ-Tubulin complexes interact with SUN in telomere positioning in yeast [88]. There are data indicating functions for γ-tubulin [61,68] and for SUN proteins [86,87] in DNA repair and in the chromatin remodeling. 

Among the proteins copurified with γ-tubulin from Arabidopsis extracts, we identified GIP proteins (Appendix A). Proteins GCP8/Mozart2 and Mozart1 were described as novel components of γTuRC, however, they did not show sequence homology with any other GCPs [102] and their role with γTuRC is only partially characterized. GIP proteins localize with the outer nuclear envelope, which is a site of acentrosomal microtubule nucleation in plants, and are also present in the nuclei beneath the inner nuclear membrane. The impaired function of GIP proteins causes the deformation of nuclei and disturbs the regular localization of SUN1 [103]. The nuclei of the *gip1* and *gip2* mutants were of abnormal shape with the aberrant distribution of proteins of nuclear pores and the aberrant organization of the centromeric region of chromosomes [104,105]. We found that GIPs interact with γ-tubulin either directly or indirectly. Both GIPs and γ-tubulin are involved in the organization of microtubules at the cytoplasmic side of the nuclear envelope and have functions in the nuclei. Therefore, we can expect that the interaction of γ-tubulin with GIPs and SUN proteins might be functional in connecting the nuclear organization with microtubular dynamics. Further experiments focused on uncovering the possible link of γ-tubulin to CRWN proteins—plant equivalents of lamins interacting with SUN proteins [97]—will provide better insight into the γ-tubulin activities with SUN/LINC in plant cells.

## 7. γ-Tubulin and Mechanotransduction

Cells subjected to mechanical cues and mechanical signals are critical for the proper development and response to environmental stress in all prokaryotic and eukaryotic organisms including animals and plants. Mechanical stimuli are transmitted from the extracellular matrix to membranes and through the microtubular and actin cytoskeleton to the nucleus. SUN1 and SUN2 are part of mechanotransduction and nuclear-cytoplasmic communication; for details follow references reviewed in Reference [106]. In animal cells, SUN1 is more tightly bound to the lamina than SUN2 and is required for the positioning of the nucleus based on the microtubular cytoskeleton, while SUN2 is required for actin-mediated nuclear movement [85]. Active forces from the cytoskeleton are transferred through the nuclear envelope to the rigid lamin nucleoskeleton and, together, regulate the nuclear and chromatin processes. Chromatin remodeling is important for the control of transcription, epigenetic status, and genome integrity. For example, the Polycomb mechanosensory pathway is important for H3K27me3-mediated silencing and morphogenesis in epidermal cells, preventing unscheduled proliferation and differentiation [107]. Mechanotransduction is responsible for laminopathy-based premature aging [108].

Integrins of animal cells are not present in plants. Instead, plant cells are equipped with the cell wall. However, the microtubular and actin cytoskeleton and SUN proteins are conserved. The nuclear envelope is suggested to be important in mechanosensing transduction to the nucleus, with SUN proteins, GIPs, and CRWN proteins being the potential components of the pathway [106]. 

Mechanosensing-like channels (MSL) may rapidly depolarize the membrane or alter the turgor pressure of plant cells in response to mechanical force [109]. Data on the MSL10 function in cell death induction suggest the broader cellular role for the MSL protein family [103]. The mechanosensitive channel of small conductance-like 6 (MSL6, At1g78610) was among the proteins identified with γ-tubulin as bait in our Y2H screen, performed as described before [110]. The interaction was confirmed under the most stringent conditions after the small-scale co-transformation of the bait γ-tubulin with the Arabidopsis cDNA library [111]. Blast analyses of the MSL6 protein sequence (with BLOSUM62) [112] showed homologs in plants and fungi but not in metazoa. Only limited data on the function of MSL6 are available. The protein is phosphorylated by calcium-dependent protein kinases (CPK1 and CPK34 [113]) and it was identified to be among plasma membrane-associated proteins differentially phosphorylated after flg22/xylanase elicitation [114]. Cortical microtubules are reoriented in response to mechanical stress through the action of microtubule-associated proteins [115]. γ-Tubulin localizes at the plant plasma membrane and with cortical microtubules and its depletion results in the microtubule randomization [19]. MSL6 protein was immunopurified with nucleoporin Rae1 in the Arabidopsis [116] and the nuclear membrane is therefore a potential site of the γ-tubulin/MSL6 interaction. The Rae1 protein, an mRNA export factor conserved in eukaryotes, is associated with the nuclear pore complex in animals [117] and it is also enriched at the nuclear periphery in tobacco cells [118]. As discussed above, γ-tubulin interacts with SUN proteins, regulates together with the transcription factor E2F1 gene expression, and has an important role with microtubules. Therefore, γ-tubulin role in mechanotransduction might be expected. However, the contribution of the interaction of the MSL6 protein with γ-tubulin to the mechanosensing in plants is only hypothetical under the current state of knowledge.

## 8. Concluding Remarks

Principles of microtubule nucleation either from centrosomes or from non-centrosomal sites are remarkably conserved in eukaryotes. This is consistent with the specialization of γ-tubulin for the microtubule nucleation early in the evolution of eukaryotic tubulins. γ-Tubulin belongs to eukaryotic tubulins and is phylogenetically close to β-tubulins, bacterial BtubB and also to artubulins (which are suggested to be direct evolutionary ancestors of the eukaryotic tubulins [3]). In accordance with phylogenetic data, it was found that γ-tubulin preserves the characteristics of both prokaryotic and eukaryotic tubulins such as tolerating a broad range of conditions for filament formation and the requirement of chaperone folding, respectively. Experimental data have recently proven that human and plant γ-tubulin preserve the ability of prokaryotic tubulin to polymerize filaments that bundle through lateral interaction [2,45,51] and, thus, confirmed the homology modeling-based prediction of γ-tubulin polymerization [47]. 

A broad distribution of γ-tubulin in several cell compartments, interaction with other cytoskeletal systems, and the number of protein interactions along with the ability to assemble filaments suggest that scaffolding or sequestration functions for γ-tubulin might exist in analogy to the filament forming prokaryotic tubulins. As there is a growing amount of evidence on the filament-forming proteins in prokaryotes, a broader definition of the cytoskeleton was proposed: “all cytoplasmic protein filaments and their superstructures that either move or scaffold (stabilize, or position or recruit) material within the cell” [52]. The expanding field of bacterial and archeal cytoskeletal filaments and data on their higher order arrays and their diverse functions in the cells are reviewed in Reference [119]. The properties of γ-tubulin discussed in our review classify γ-tubulin as a cytoskeletal protein. γ-Tubulin filaments are present with membranes in nuclei and also on microtubules [2]. However, with a still-missing mechanistic understanding of the filament structure and interactions, we can only hypothesize whether the formation of γ-tubulin filaments in vivo may occur on a supporting matrix of membranes, DNA or other filaments via the collaborative filament formation defined in Reference [53]. 

**Figure 2:**
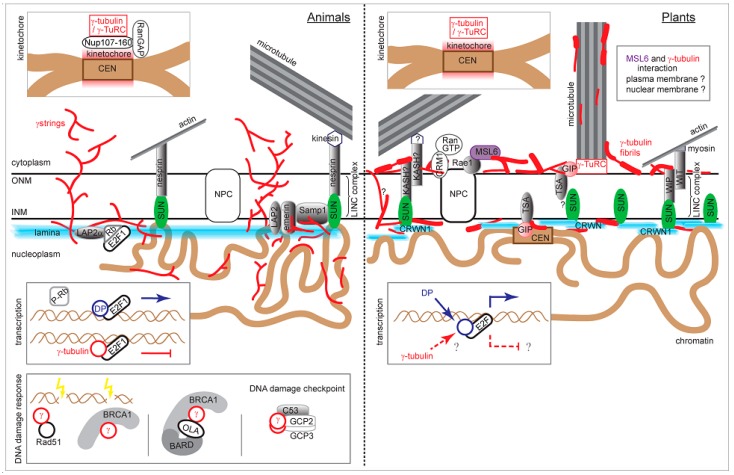
The graphical summary of nuclear γ-tubulin interactions and functions in animal and plant cells. Particular sections are based on published data or modified according to the cited references: γ-strings [41], γ-tubulin fibrils [2], LINC complexes [96,106], lamina interactions and INM integral proteins [77], emerin and Samp1 [100,101], SUN protein interactions [82,97], CRWN (former LINC) proteins [97,121,122], GIP [105], Rae1 and MSL6 [123], kinetochore [22,23], E2F regulation [69,77,124], DNA damage response [61,64,125]. ONM: outer nuclear membrane; INM: inner nuclear membrane; NPC: nuclear pore complex; CEN: centromere. Not to scale.

As far as we know, nuclear γ-tubulin was demonstrated in acentrosomal plant cells [60] for the first time and the presence of γ-tubulin in nuclei and its functions in nuclear processes are now also generally accepted in animals. Recent data on the interaction of γ-tubulin with lamin and its function in the nuclear organization [41] expanded the cohort of γ-tubulin nuclear functions (Figure 2). We found that γ-tubulin interacts with conserved SUN proteins of the LINC complex in plants where lamins are not encoded in genomes. The comparative studies of γ-tubulin nuclear functions in lamin-equipped animal and lamin-free plant cells would help us understand a γ-tubulin role in the spatial and temporal coupling of microtubular dynamics and nuclear integrity.

The mechanistic understanding of the structure of γ-tubulin filaments is needed to get better insight into the molecular biology of γ-tubulin and its functions. Cryo-EM with high resolution and helical reconstitution was used to solve the structure of prokaryotic filaments [120] and, similarly, the atomic model of γ-tubulin filaments, including their lateral interaction in higher order assemblies, is necessary. The reconstitution of filaments in vitro and super-resolution microscopy of fluorescently labeled proteins or total internal reflection microscopy TIRF combined with electron microscopy TEM will also help in the understanding of the building principles of γ-tubulin linear polymers. However, light microscopy has its limitation, such as the use of fluorescent tags which might be, due to sterical hindrance, potentially disruptive. Additionally, resolving power, which is lower than the dimension of a single γ-tubulin filament, presents a limitation of light microscopy. Cryoelectron tomography in cells allows for the visualization of subcellular structures in a native state with a resolution of 4 nm. This technique will be essential for understanding γ-tubulin filaments and their higher-order assemblies in the cellular context. Due to the fast progress in advanced microscopy techniques, the studies of γ-tubulin cellular functions suggested here are timely. 

## Figures and Tables

**Figure A1:**
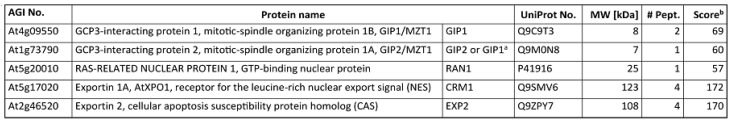
The list of selected proteins copurified with γ-tubulin from Arabidopsis thaliana. Proteins were identified by LC MALDI-TOF MS/MS [2]. The number of matching peptides and the total MASCOT score for each protein are shown. ^a^ The only peptide identified matches both GIP1 and GIP2 protein sequences. ^b^ Sum of MASCOT scores of all identified peptides.

## Abbreviations

CM-SR	confocal laser scanning microscopy equipped with a super-resolution module
LINC	Linker of Nucleoskeleton and Cytoskeleton

## Appendix B. Fluorescence Microscopy

Root tips of *Arabidopsis thaliana* ecotype Columbia 0 (Col-0) were used for immunolabeling. Slide preparation of squashed root tips was performed according to Reference [126]. Mouse monoclonal anti-γ-tubulin TU32 ([127], 1:5), anti-SUN1,2 (Agrisera, 1:600) and anti-α-tubulin (Abcam, 1:2000) antibodies were used in combination with Alexa Fluor 488- and Alexa Fluor 594-conjugated anti-mouse and anti-rabbit antibodies (Jackson ImmunoResearch Laboratories, 1:200). Chromatin was stained with DAPI. Cells were analyzed by Olympus IX-81 FV-1000 confocal scanning imaging system equipped with the super-resolution Olympus FV-OSR software module with an oil immersion objective x100/1.45. For details of confocal laser scanning microscopy, see Reference [2]. Images were deconvolved using Huygens (Scientific Volume Imaging, The Netherlands) and analyzed in Fiji.

Immunofluorescence labeling for STED microscopy was performed according to Reference [2]. Secondary antibodies Abberior STAR RED (1:200) for γ-tubulin and Aberrior STAR 580 (1:200) for α-tubulin were used. Images were obtained by pulsed stimulated emission (pulse-STED, 775 nm depletion laser) super-resolution microscope Leica TCS SP8 STED 3X. For the stimulated depletion, 90% intensity of 775 nm depletion laser was used. Deconvolution was done by Huygens Professional version 16.05 (Scientific Volume Imaging, The Netherlands). For more information, see Reference [2].
